# A 3D-Printed Knee Wearable Goniometer with a Mobile-App Interface for Measuring Range of Motion and Monitoring Activities

**DOI:** 10.3390/s22030763

**Published:** 2022-01-20

**Authors:** Bryan Rivera, Consuelo Cano, Israel Luis, Dante A. Elias

**Affiliations:** Laboratory of Biomechanics and Applied Robotics, Pontificia Universidad Católica del Perú, Lima 15088, Peru; consuelo.cano@pucp.edu.pe (C.C.); israel.luis@pucp.pe (I.L.); delias@pucp.pe (D.A.E.)

**Keywords:** wearable, goniometer, Hall-effect sensor, algorithm

## Abstract

Wearable technology has been developed in recent years to monitor biomechanical variables in less restricted environments and in a more affordable way than optical motion capture systems. This paper proposes the development of a 3D printed knee wearable goniometer that uses a Hall-effect sensor to measure the knee flexion angle, which works with a mobile app that shows the angle in real-time as well as the activity the user is performing (standing, sitting, or walking). Detection of the activity is done through an algorithm that uses the knee angle and angular speeds as inputs. The measurements of the wearable are compared with a commercial goniometer, and, with the Aktos-t system, a commercial motion capture system based on inertial sensors, at three speeds of gait (4.0 km/h, 4.5 km/h, and 5.0 km/h) in nine participants. Specifically, the four differences between maximum and minimum peaks in the gait cycle, starting with heel-strike, were compared by using the mean absolute error, which was between 2.46 and 12.49 on average. In addition, the algorithm was able to predict the three activities during online testing in one participant and detected on average 94.66% of the gait cycles performed by the participants during offline testing.

## 1. Introduction

The maneuverability of a joint within its Range of Motion (ROM) is an indicator of functional performance in the human body. This functional performance can be used for assessment in rehabilitation, identifying pathologies, etc. Passive ROM (PROM) involves when an external force moves the joint, while Active ROM (AROM) involves when the movement occurs due to contraction of the muscles. For measuring the PROM, medical professionals commonly use goniometers [1], while, during AROM, motion capture systems able to measure the functional ROM variables in real-time must be necessary. Optical motion systems involve cameras and markers; they are the gold standard for measuring kinematic variables but involve costs and time to get ready. Due to this limitation, the use of wearable technology as motion capture systems has been promoted [2,3,4,5,6], as they have the potential to measure kinematic parameters during daily activities and in less restricted environments due to their portability, allowing continuous monitoring of the joint [4,5].

Companies and academic institutions have been developing wearables for motion capture purposes. In the case of companies, Biometrics Ltd. developed flexible goniometers by using resistive transducers, and their goniometer can measure the angles of the knee, ankle, and wrist [7]. Other examples are the Aktos-t system developed by MYON [8] and the MVN Analyze system [9] of XSens (https://www.xsens.com/, accessed on 15 August 2021); these systems use Inertial Measurement Units (IMUs) and have their specialized software. In the case of academic institutions, diverse systems using potentiometers [10], IMUs [11,12], Hall-effect sensors [13], and knitted piezoresistive fabric technology [14] were developed.

Wearable technology can be integrated with algorithms, applied in real-time or after the data is collected, into portable systems that could identify biomechanical patterns. Examples of real-time applications from the literature include using machine learning to measure biomechanical risk during lifting tasks [15] and using state machine [16], classification tree [17], and fuzzy computational [18] to detect events of the gait cycle. The wearables from these studies used IMUs to obtain the angles of the articulations, and some of them presented other sensors such as insole pressure sensors [17] and force plates [18], which were used to obtain the values of the ground reaction forces. In a systematic review, Adesida et al. found out that IMUs are the most used sensors for the development of wearables to assess sports biomechanics compared to other technologies as flex sensors and magnetic sensors [6]. These findings support the idea that IMUs are the preferred choice that researchers and companies choose for the development of wearables for motion capture purposes. However, one problem of IMUs is the drift, an error that occurs due to the gyroscope and increases over time [19], and one reason why the commercial solutions based on IMUs are expensive is due to the engineering process involved to reduce this drift, for instance, through sensor fusion [20]. In addition, IMUs are more useful when it is necessary to measure the three angles from a 3D space, so, for measurement of a single axis such as knee flexion/extension, an IMU-based solution may be too complex. Therefore, it opens the opportunity to explore other technologies in the field of rotatory sensors, which do not present the drift error.

Wearables are made of different materials, and the examples based on IMUs [11,12,15,16,17,18] employed straps are made of textiles. For sensors that need to be aligned with the joint axis of rotation, a mechanical structure is necessary. For instance, a goniometer that worked through a Hall-effect sensor employed a structure composed of bars made of duraluminium (13). Still, it is important to consider the use of lightweight materials such as Acrylonitrile Butadiene Styrene (ABS) or Polylactide (PLA), which can be done through 3D printing. Even though these materials do not present high stiffness as metals, it is not always necessary for the loads to which the goniometer is subjected as the function of these devices is just to take data. A wearable following this approach was developed by the authors of this article [21] as the first model of the goniometer; this was designed to measure the knee flexion angle and the ankle dorsiflexion/plantarflexion and inversion/eversion; however, it was not tested in participants while performing the activity of walking.

Wearables have been promoted in the literature for applications that involve biomechanical analysis, such as sports, rehabilitation, and clinical diagnosis [22], as they could be useful for monitoring the user’s progress or detecting abnormalities in gait patterns [22]. In addition, the data collected from wearables can be used for classification algorithms or statistical testing, techniques that could help differentiate biomechanical patterns between pathological and control groups. Moreover, compared to camera-based motion capture systems, wearables can be used during extended periods while doing daily activities [23], providing biomechanical data that could be useful for researchers and clinicians.

The present article proposes the development and preliminary testing of a 3D-printing knee wearable goniometer for AROM assessment as a continuation of the previous design made by the authors [21]. The wearable uses only one Hall effect sensor to measure knee flexion/extension angle and send this information to an Android device (https://www.android.com/, accessed on 1 August 2021) that shows the angle in real-time and the name of activity the user is performing: sitting, standing, or walking; which are detected through an algorithm. In addition, the measurements of the wearable were compared with two systems. First, it was tested if it could measure the same values that a commercial goniometer. Secondly, its measurements were compared to the ones provided with an Aktos-t system while participants walked at three different speeds wearing both systems. The wearable presents one degree of freedom and has specific dimensions; therefore, improvements are expected as the knee joint presents more than one degree of freedom [24] and personalization may be needed for specific sizes of subjects. Moreover, the objective of the comparisons performed in this study is to get an idea of which aspects of the wearable need improvement. The purpose of the final design of the wearable is to be considered as an alternative to assess and monitor long-time activities in the healthcare and industrial sector as a quick, affordable, and specialized tool; and to motivate the integration of 3D-printing wearable technology and algorithms for movement analysis by using a lower quantity of sensors.

## 2. Materials and Methods

### 2.1. Wearable Description

The development of the wearable involved mechanical design, electronic design, and programming. As mentioned in the previous section, this wearable of this study is an improvement of a previous one developed by the authors [21]. The improvements of the current wearable compared to the older are its compacter design, the implementation of an algorithm for activity recognition, and transmission of the joint angle to an Android device.

#### 2.1.1. Mechanical Design

**Figure 1:**
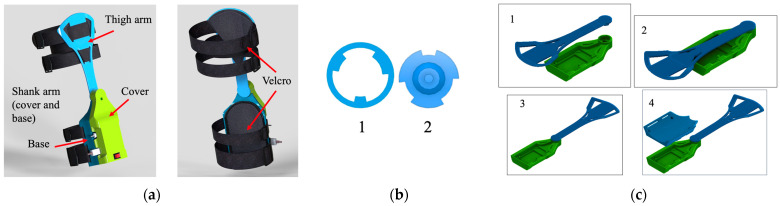
(**a**) CAD model of the knee wearable; (**b**) mechanical attachment pattern: 1—shank arm pattern, 2—thigh arm pattern; (**c**) assembly process of the wearable goniometer: (**1**,**2**) the base and the thigh arm are connected in the specific position (**3**,**4**) and the thigh arm is rotated 180° to allow the placement of the cover.

**Figure 2:**
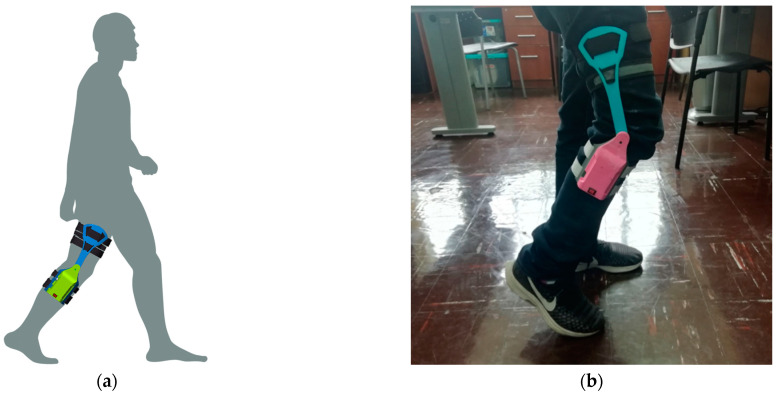
(**a**) 3D model of a person using the wearable; (**b**) a user wearing the wearable goniometer.

The wearable presents one degree of freedom with two arms, one for the shank and another for the thigh as shown in Figure 1a. For the material’s features, it was considered simple to manufacture, lightweight, and low-cost; therefore, the pieces were 3D-printed in ABS (Crear 4d SAC., Lima, Peru) using Ultimaker 3 Extended, although it can also be printed with PLA or other similar materials with the characteristics explained. The sensor and the electronic components are embedded inside the shank arm structure, which is composed of the cover and the base as shown in Figure 1a. The thigh arm is assembled to the shank arm by using a novel mechanism that was developed for the first design [21]. This mechanism, shown in Figure 1b, allows the wearable to be assembled and disassembled when the thigh arm and shank arm form an angle of 180° (Figure 1c), which is not possible for human motion; therefore, the wearable will not disassemble when the user is wearing it. The rotation axis of the wearable is aligned to the one of the knee flexion/extension, and to place the wearable on the user, Velcro combined with straps and elastic bands were used for each arm, so they attach to the lower limb of the user as shown in Figure 2a,b.

#### 2.1.2. Electronic Design

The magnetic rotatory encoder AS5048B (AMS AG) was used for joint angle measurements. The data are read and processed by the microcontroller Atmega328 (Atmel) and sent via Bluetooth communication with a frequency of 100 Hz to an Android device or a personal computer. This frequency was considered because it is enough for kinematic variables during gait analysis as it allows an appreciation of how the variables change during one gait cycle. The microcontroller and the Bluetooth are integrated into the commercial electronic board Beetle Bee, which was developed by DFROBOT. The system is energized by a Lithium-Ion Polymer (LiPo) 3.7 V 1200 mAh battery, whose voltage is amplified and regulated to 5 V through the Powerboost 1000 C (Adafruit Industries), which also allows the recharging of the battery. To avoid the use of cables, the components were integrated into an electronic board made of fiberglass.

**Figure 3:**
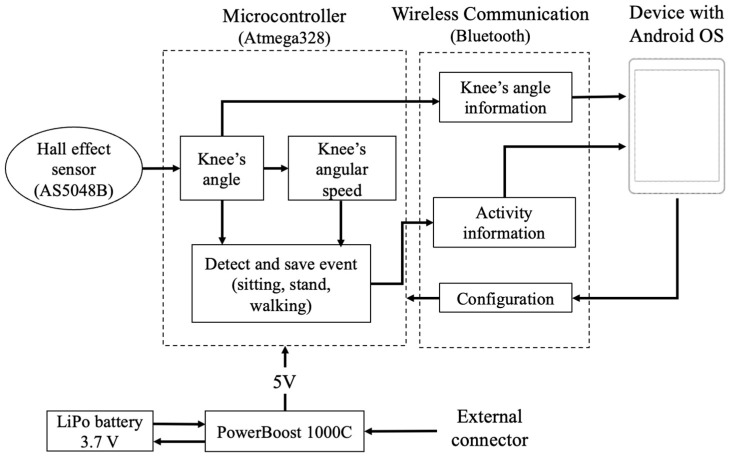
Interaction of the components of the wearable goniometer.

Figure 3 shows the interaction of the elements; it can be appreciated that the angular speed is calculated from the angle to predict the user’s activity, and this variable is obtained from the knee angle and the sample time.

#### 2.1.3. Mobile Application Interface

An application in Android OS was developed through Android Studio to display the knee angle and activity name in real time. This app allows the setup of the knee which will be measured (left or right) and zero position (knee fully extended). Once these parameters are configured, the app shows the angle and the activity in real time. The configuration can be reset at any moment.

### 2.2. Algorithm for Activity Detection

The algorithm for activity detection (standing, sitting, and walking) is based in a finite state machine that uses the angular position (θ_n_) and angular speed (ω_n_) of the knee joint. The angular position is obtained by applying a median filter to the eight last angles provided by the sensor, and the angular speed is obtained by dividing the difference of two consecutive filtered positions (θ_n_−θ_n−1_) by the sample time Δt = 10 ms.

The finite state machine consists of five states (S_0_, S_1_, S_2_, S_3_, S_4_), which each of them representing a pattern and situation. These states were used to differentiate between standing, sitting, and walking. The system is initially on the state S_0_, which means that the user is not doing an activity that involves movement; during the sequence of states S_1_, S_2_, S_3_ and S_4_, the user is doing the activity of walking.

The detection of standing and sitting activity occurs only during the state S_0_. During this state, the angles provided during the last second (100 samples) are compared with the angles 20° and 70°. If the angles are less than 20°, the user is standing; on the other hand, if the angle is more than 70°, the user is sitting. The angular speed was not considered for the detection of these activities.

**Figure 4:**
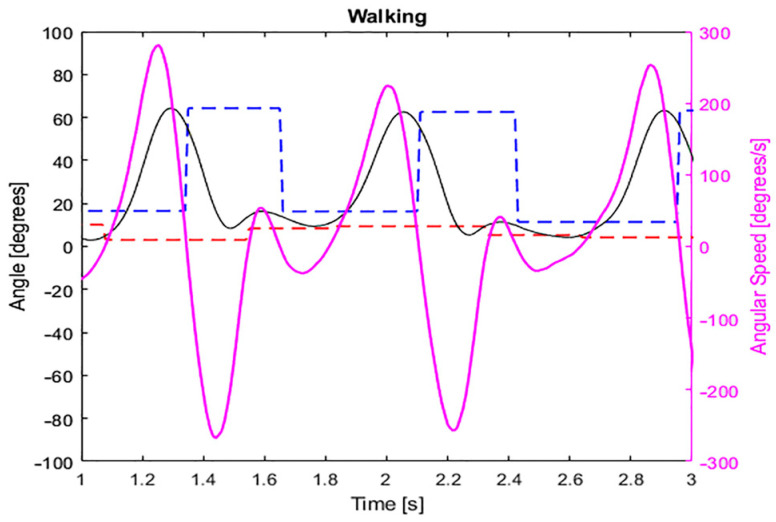
Knee’s angle (black continuous), angular speed (pink continuous), maximum (blue dashed line) and minimum angle (red dashed line) in the activity of walking.

For the sequence of states S_1_, S_2_, S_3_, and S_4_, the maximum and minimum peaks of knee flexion angle during the gait cycle were analyzed. The change of the knee angle during one cycle of walking activity presents four concavities, two concaves up and two concaves down, which represent the peaks of extension and flexion, respectively. The values during these situations can be considered as local maximum for concave down, or local minimum for concave up. Therefore, the local maximum (θ_max_), and local minimum (θ_min_) in one cycle were analyzed for the detection of the activity and transition of states. To obtain those values, the system evaluates the sign of the angular speed (ω_n_) and compares the current angular position (θ_n_) with the two previous values (θ_n−1_, θ_n−2_). For instance, to find θ_max_, the sign of ω_n_ should be positive, and θ_n_ should be increasing (θ_n_ > θ_n−1_); once it stops increasing (θ_n+1_ < θ_n_), the final value of θ_n_ is assigned to θ_max_. Similarly, to find θ_min_, ω_n_ should be negative and θ_n_ should be decreasing (θ_n_ < θ_n−1_), and the value of θ_n_ is assigned to θ_min_ when θ_n+1_ > θ_n_. Figure 4 shows the results of the algorithm applied to a set of data. It can be appreciated that the values of θ_max_ (blue dashed line) and θ_min_ (red dashed line) do not change until another local maximum/minimum is detected.

**Figure 5:**
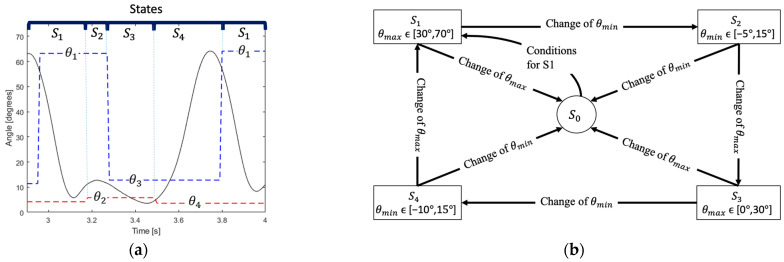
(**a**) States and maximum/minimum angles during the activity of walking; (**b**) sequence of states S_0_, S_1_, S_2_, S_3_, and S_4_ with their respective condition for change, the detection of the walking activity occurs when a transition from S_4_ to S_1_ occurs.

The initiation of movement involves the transition from S_0_ to S_1_, which is evaluated through θ_max_, θ_min_, and ω_n_. Firstly, it is compared if ω_n_ is higher than 120°/s. Then, it is evaluated if θ_max_ has changed while θ_min_ is kept constant. The ranges in which θ_max_ and θ_min_ are compared are [30°, 70°] and [−5°, 15°], respectively. Once these three conditions are satisfied, the state changes to S_1_. This logic is represented by “Conditions for S1” in Figure 5b.

For detection of the walking activity, the changes of θ_max_ and θ_min_ are evaluated during the sequence of states S_1_, S_2_, S_3_, and S_4_. The sequence of angles is defined as θ_1_, θ_2_, θ_3_ and θ_4_, which should stay in the range of [30°, 70°], [−5°, 15°], [0°, 30°], and [−10°,15°], respectively, in order to complete a cycle of walking. These values were set after observing the data obtained from the wearable. The angles θ_1_ and θ_3_ are maximum angles (peak in flexion), while θ_2_ and θ_4_ are minimum angles (peak in extension). Figure 5a shows how the states change during the activity of walking, and the transition from one state to another occurs when there is a change in the value of θ_max_ or θ_min_ depending on the state.

As observed in Figure 5b, the sequence of states is evaluated by specific conditions, and, if one of the conditions is not satisfied, the wrong angle changed or the angle is not in the range, the system returns to the state S_0_. In addition, if the system stays in one state of movement (S_1_, S_2_, S_3_, or S_4_) for more than 2 s, it is returned to the state S_0_. The detection of one gait cycle is done by the transition from S_4_ to S_1_.

### 2.3. Preliminary Tests

#### 2.3.1. Participants

For the testing of AROM, nine healthy participants (7 males, 2 females, 67.39 ± 10.83 kg, 163.44 ± 5.85 cm) used the wearable. They were asked if they did not present any gait disorder; however, no clinical test to confirm any pathology that could affect gait was taken. The participants were considered due to their different heights and weights, as it could give an idea of how the error changed during AROM, and how the algorithm could detect the activity of walking with the current design of the wearable.

#### 2.3.2. Comparison with a Commercial Goniometer

**Figure 6:**
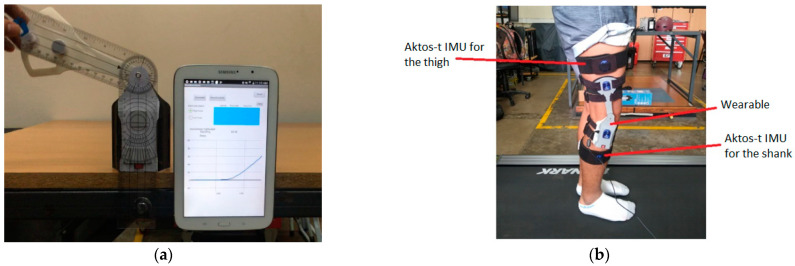
(**a**) Set up for the comparison of the wearable and the commercial goniometer; (**b**) set up for testing of the AROM. The wearable and the Aktos-t are worn by the subject.

A commercial goniometer was placed parallel to the wearable as shown in Figure 6a. Double contact tape was used to keep both goniometers aligned, allowing the goniometer and wearable to move together. The information of the wearable, which is shown on the Android device, is compared to the measurement of the goniometer. For this test, the information of the wearable was sent to the Android device, so it was not stored.

#### 2.3.3. Testing of AROM Measurement

The participants were asked to wear the wearable while walking in a treadmill at 4 km/h, 4.5 km/h, and 5 km/h for 20 s. All participants performed the gait activity without the need of any assistive device. The data were transmitted to a computer via serial communication at 100 Hz and read and stored through a program developed on Matlab (MathWorks, Inc., Natick, MA, USA). The data of the wearable were compared to the data provided by an Aktos-t IMU system (Myon AG), in which one IMU was placed in the thigh and another in the shank of the subjects as shown in Figure 6b. This Aktos-t system has been tested for gait analysis by Bessone et al. [25]; they compared it with a Vicon optoelectronic motion capture system (Vicon Motion Systems, Oxford, UK) and compared the root mean squared error (RMSE), in which they found that the accuracy of measuring the knee was between acceptable (RMSE < 5°) and tolerable (RMSE < 10°).

The sampling frequency of the Aktos-t system was 200 Hz. To avoid sliding of the wearable in the users, they were asked to wear short or thigh pants depending on their preference.

**Figure 7:**
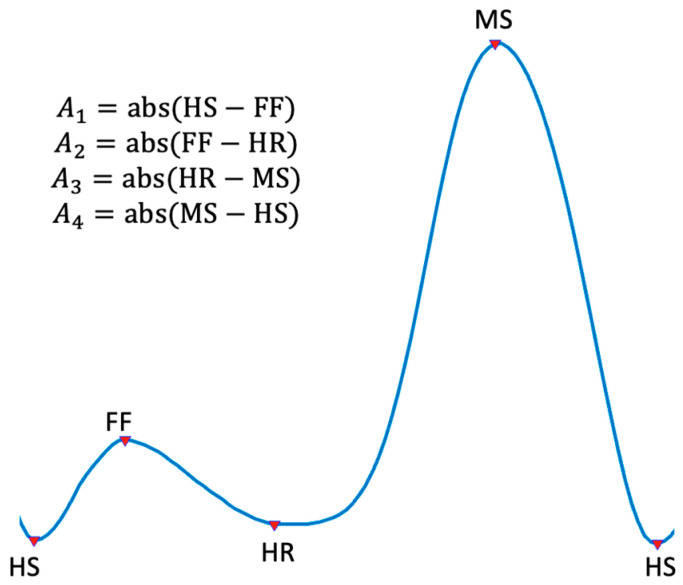
Location of the peaks in the gait cycle and calculation of each difference (A_1_, A_2_, A_3_, A_4_). There are two peaks during flexion: Foot-flat (FF) and Mid-Swing (MS); and two peaks during extension: Heel-Strike (HS) and Heel-Raise (HR).

To compare the AROM measurements from the wearable and the Aktos-t system, the differences between the peaks of knee flexion and extension obtained by both systems were compared. This approach was used to avoid the effect of different systems of references used during the measurements in each system. During the gait cycle, there are two peaks of flexion: foot-flat and mid-swing, and two peaks of extension: heel-raise and heel-strike, which gives a total of four differences for one cycle. The four differences will be tagged as A1, A2, A3, and A4, and they are calculated as the absolute difference of two peaks that are next to each other. In that sense, A1 will be the absolute difference between heel-strike and foot-flat, A2 the absolute difference between foot-flat and heel-raise, A3 the absolute difference between heel-raise and mid-swing, and A4 the difference between mid-swing and heel-strike of the next cycle. An illustration of the four peaks of the gait cycle and how the differences are calculated is shown in Figure 7.

For each user, Mean Absolute Error (MAE) for each difference during the first six gait cycles was calculated. Therefore, a total of four MAE for one user walking at a specific speed was obtained. Then, for each difference and speed, the average of all the user’s MAE was calculated to get an estimation of the error of the wearable. In addition, to observe the variability for the error, the Mean Absolute Deviation (MAD) was calculated for each user in different speeds, similarly to the logic used for the MAE.

### 2.4. Testing of Algorithms for Activity Detection

#### 2.4.1. Online Testing

To test the algorithm in real-time or online, one person (male, 54.9 kg, 1.63 cm) performed the activities of standing, sitting, and walking. The activity performed by the user was shown in the Android device.

#### 2.4.2. Offline Testing

Detection of the activity of walking was tested in the data acquired during AROM comparison. During the procedure, it was found that the offset in some users was set incorrectly, as the knee angles were around 100°–360° when it should be zero. Therefore, the first angle of the analyzed data was set up, which represents heel-strike, to zero, adapting the rest of each participant curve to the new system of reference. Similarly, to the AROM test, the algorithm was only tested in the first six gait cycles.

To evaluate how well the algorithm detected the activity of walking, the efficiency was obtained by dividing the number of detected gait cycles by the total of cycles that passed through the algorithm. It is important to mention that a gait cycle was detected each time a transition from S4 to S1 occurred.

## 3. Results

### 3.1. Comparison with a Commercial Goniometer

**Figure 8:**
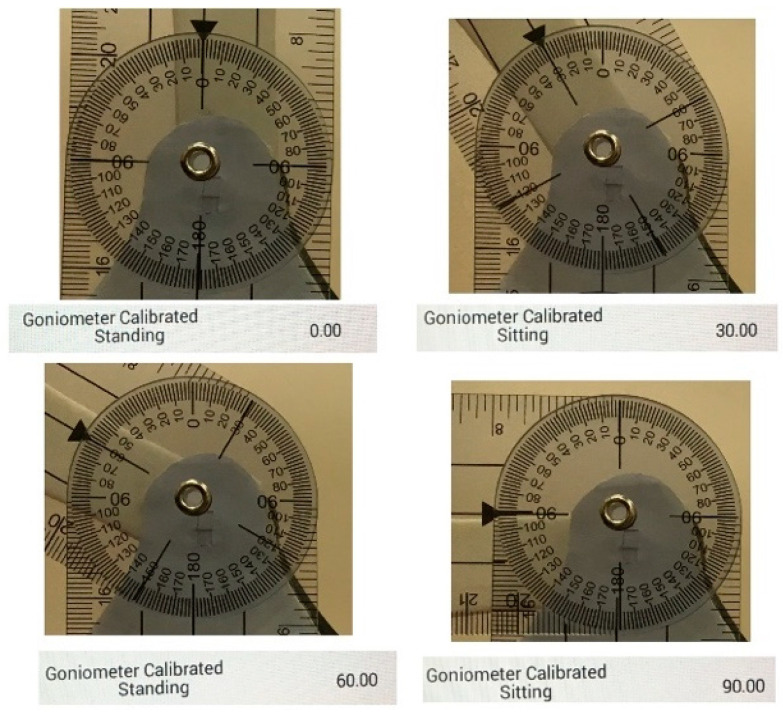
Results of the comparison between wearable and commercial goniometer.

Four measurements (0°, 30°, 60° and 90°) were taken from the wearable and the goniometer. Both values were similar with a precision of 1°, which is the one presented by the commercial goniometer. Figure 8 shows the comparison for the four angles, and the value of the wearable is shown in the Android app.

### 3.2. AROM Measurement

The variation between the wearable and the Aktos-t IMUs was higher when comparing the angle between mid-swing and heel-strike (A_4_). The minor difference was observed when comparing the angle between foot-flat and heel rise (A_2_). Table 1 shows the averages and standard deviations of the MAE for different walking speeds.

MAE shows that the error of the wearable varies for different subjects. For instance, subjects 1, 4, 5, and 8, as shown in Table 2, presented an error lower than 10°, while the rest of the subjects presented errors around 10°–20°, specifically for the A_4_. In addition, the MAD shows that there is variability in the error less than 2° as observed in Table 2. Details about the MAE and MAD of each subject at different speeds can be observed in Table A1 and Table A2, respectively, both located in Appendix A.

### 3.3. Algorithm

#### 3.3.1. Online Testing

**Figure 9:**
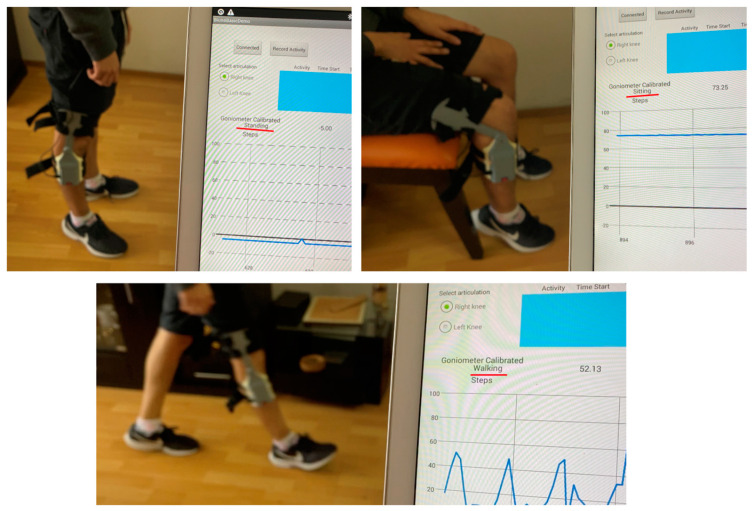
Results of the algorithm in real-time testing, and the activity shown by the app is underlined with red. **Top left**: Standing. **Top right**: sitting. **Bottom**: walking.

Figure 9 shows the detection of standing, sitting, and walking in real time. During the tests, the activity “walking” was commonly detected after the user performed the third step, which means it may be necessary to perform adjustments to reduce the communication time between the wearable and the Android device. However, once this activity was detected, it was shown on the Android device until the person stopped walking.

#### 3.3.2. Offline Testing

The algorithm for walking was tested in the data obtained for the comparison of AROM. As observed in Table 3, the algorithm detected most of the cycles with an accuracy of 94%, 96%, and 94% for the speeds of 4.0 km/h, 4.5 km/h, and 5 km/h, respectively.

## 4. Discussion

A novel wearable made of 3D printing material was developed for measuring the knee AROM and detecting activities. This wearable attaches to different sizes of the lower limbs of the user through a novel textile made of Velcro straps and elastic bands. A user interface was implemented, which allows the observation of the angle and the activity in real time.

During the comparison between the wearable and the commercial goniometer, there was no difference in the measurements between both devices. Therefore, the wearable can measure similar values that a commercial goniometer has. In addition, during the comparisons, the values of the wearable ended with 0.00°, suggesting that the device can provide an accuracy of two decimals. During the comparison of AROM, the greatest and smaller errors occurred during the comparisons of A_4_ and A_2_, respectively, which may suggest that the error increases with the movement of the angle. One possible cause might be the misalignment between the wearable and the knee joint, which may occur due to the translation, sliding, and rotation of the femur during the movement of flexion. For instance, the literature suggests that the knee presents two centers of rotation located in the femur [24]: Extension Facet Center (EFC) and Flexion Facet Center (FFC), which changes depending on the knee flexion angle. For instance, previous studies have demonstrated that the FFC moves around 30 mm on the lateral side and 10 mm on the medial side during flexion [26], which suggests that the knee rotates around a vertical axis located in the medial side during the movement of flexion [24]. Moreover, it was found that, during the activity of walking, this vertical axis is located in the lateral side, suggesting that the rotation of the knee may change depending on the performed activity [27]. These findings suggest that a more specialized mechanism may be necessary to adapt to the movement of the knee flexion axis during the activity of walking that may be considered for future designs of the wearable.

Moreover, the order of the errors from smaller to greatest is A_2_-A_3_-A_1_-A_4_, which shows that, even though A_3_ is greater than A_1_, it presented a smaller error. This suggests that the error does not only depend on the amplitude of the ROM but other factors. One factor why the error is greater in A_1_ and A_4_ may be the impact during heel-strike, which occurs between the phases in which both values are taken (see Figure 7). Previous literature has stated that the legs absorb most of the vibrations during heel-strike [28]; in addition, part of the vibrations are absorbed by the meniscus of the knee [29]. Therefore, it is possible that these vibrations misaligned the wearable and increased the error of the angle. Furthermore, this vibration might have increased as the subjects were not wearing shoes during the trials, and previous research has found that the loading rate of the ground reaction forces increased when the participants were not wearing shoes [30]. This suggests that future designs should consider mechanisms for the compensation of vibrations and opens the possibility to study the effect of vibrations in the system.

The error varied between different participants even though a design of straps and elastic bands was considered to adapt the connection of the wearable to a different radius of thigh and shank. Therefore, it may also be necessary to adapt the design of the structure for different sizes of participants, in a way that it adapts to the geometry of their leg or anthropometric measurements. It is also important to point out that, during the design, the wearable was continuously tested in a male ectomorph subject whose characteristics were 54.9 kg and 1.63 cm; therefore, it may be possible that the wearable measures better in subjects with similar size.

The error presented variability smaller than 2°, as observed in the MAD. This variability may be related to the displacement of the offset during trials, which may occur due to sensor drift (Aktos-t IMUs and wearable Hall effect sensor) or the misalignment of the wearable during each step of the trial. However, as the time of the trial is short, it is possible that the main reason of displacement is misalignment.

The activity of walking was detected, during online and offline tests, by using the proposed finite state machine algorithm. The algorithm used a large range of angles in the conditionals because the reliability of the device still needs to be assessed, a test that will be performed once the design of the wearable is improved to avoid misalignments during its use. However, the results show that the use of finite state machine algorithms to detect the activity of walking is an adequate alternative. Once the design is validated, this algorithm can be integrated with machine learning techniques to personalize the range of angles for each subject. Moreover, detection of sitting and standing was tested in just one person. Nevertheless, as their logic of detection in the algorithm just involved the comparison of angular values in a specific range; expectations are that the algorithm could detect both activities correctly in more subjects once the device presents validated reliability.

There are limitations of this study that should be acknowledged. Firstly, one design of the wearable in all participants was used, and even though the straps were designed to adapt for different sizes, it may also be necessary to take into consideration the size of the structure. This consideration should be taken in conjunction with a mechanism that could adapt to the movement of the knee flexion axis during gait. In addition, although it was checked that all participants walked without problems, it may also be necessary to take clinical tests to make sure that they did not present any disease or condition that may have affected their gait during the tests. Furthermore, the comparison was done with an Aktos-t IMU system, which, according to literature [25], presents an RMSE less than 5° and 10° measuring lower limbs activity when compared to a Vicon Motion Capture System.

Future development of the wearable is expected to save data through the Android device or the wearable controller, which would allow storage of data from daily activities without the need of being connected to a computer, giving more portability. In addition, the use of this data could allow evaluation of the evolution of treatment in patients, statistical studies which involve gait analysis in different groups, improve capacities of functional ROM through training, or evaluation of functional ROM in workers during labor activities.

## 5. Conclusions

This wearable did not present differences in its measurements during comparison to a commercial goniometer. The values in AROM presented differences compared to the values provided by the Aktos-t IMU system, and it may be necessary to consider the tibiofemoral movement and the sliding [24,26] to improve the design. Moreover, it was possible to develop an algorithm that could detect three activities (sitting, standing, and walking) by only considering the knee flexion angle and angular speed. The range of maximum/minimum values used in the algorithm was large, as the reliability of measurements still needs to be assessed. Furthermore, the technical specifications of the device serve as an illustration to develop 3D-printing wearable technology for motion assessment. Finally, this wearable serves as the first concept of one final design that does not intend to replace optical motion capture systems, but to be used as a quick assessment tool for health practitioners or researchers to provide an initial quantifiable measurement of knee flexion angle for possible diagnosis of pathologies, or monitoring gait patterns during rehabilitation, for example, in patients with knee injuries such as meniscal tears, fractures, and ligament injuries, especially in a context where the time of consultation, cost, or space is limited.

## 6. Patents

A utility model patent was granted due to the development of the wearable electrogoniometer on 31 December 2019. (N° 003709-2019/DIN-INDECOPI).

Name of patent: “*Goniómetro modular portatil con sensores de efecto Hall*”. English translation “Modular portable goniometer with Hall-effect sensors”.

## Figures and Tables

**Table 1:** Average of the MAE during the comparison of the differences calculated by the wearable and the Aktos-t system during the activity of walking. The results are shown in the format mean ± standard deviation.

	Mean Absolute Error			
Speed	A_1_	A_2_	A_3_	A_4_
4.0 km/h	7.72° ± 4.01°	2.32° ± 1.66°	5.90° ± 4.84°	12.09° ± 8.60°
4.5 km/h	6.81° ± 3.41°	2.33° ± 1.65°	5.61° ± 4.57°	11.77° ± 7.43°
5.0 km/h	7.85° ± 2.57°	2.72° ± 1.79°	6.86° ± 5.72°	13.61° ± 7.06°
Total	7.46° ± 3.28°	2.46° ± 1.65°	6.12° ± 4.90°	12.49° ± 7.47°

**Table 2:** MAE and MAD for each subject considering the differences for the three speeds (4.0 km/h, 4.5 km/h and 5 km/h).

			Mean Absolute Error				Mean Absolute Deviation			
Subject	Weight (kg)	Height (cm)	A_1_	A_2_	A_3_	A_4_	A_1_	A_2_	A_3_	A_4_
1	60	159	4.95°	1.27°	3.43°	2.57°	1.20°	0.85°	1.03°	1.17°
2	85	172	6.22°	3.40°	7.10°	12.94°	1.26°	0.93°	1.21°	1.18°
3	79	164	8.95°	5.51°	15.41°	18.93°	1.17°	0.97°	0.93°	1.02°
4	70	167	7.42°	2.84°	1.34°	5.88°	1.31°	0.95°	0.66°	1.08°
5	68	168	4.52°	1.86°	1.75°	6.55°	1.86°	1.52°	1.73°	1.87°
6	67.6	163	9.44°	3.47°	10.19°	23.02°	1.33°	1.25°	1.46°	1.44°
7	67.9	168	8.88°	1.25°	3.38°	13.33°	1.21°	0.72°	1.03°	1.66°
8	44	152	5.98°	1.16°	4.00°	8.83°	0.51°	0.62°	0.70°	1.02°
9	65	158	10.77°	1.38°	8.53°	20.35°	1.38°	0.73°	1.12°	1.66°

**Table 3:** Result of the detection of the activity of walking.

Subject	Speed		
	4.00 km/h	4.50 km/h	5.00 km/h
S1	83%	100%	100%
S2	67%	100%	100%
S3	100%	100%	100%
S4	100%	100%	100%
S5	100%	100%	100%
S6	100%	67%	67%
S7	100%	100%	100%
S8	100%	100%	83%
S9	100%	100%	100%
Average	94%	96%	94%
Total	94.66%		

## Data Availability

The data presented in this study are available on request from the corresponding author.

## Appendix A

**Table A1:** MAE of each one of the four differences at different speeds in all subjects.

			4.0 km/h				4.5 km/h				5.0 km/h			
Subject	Weight (kg)	Height (cm)	A_1_	A_2_	A_3_	A_4_	A_1_	A_2_	A_3_	A_4_	A_1_	A_2_	A_3_	A_4_
1	60	159	4.08°	1.10°	4.24°	1.28°	4.39°	1.42°	3.98°	1.92°	6.37°	1.29°	2.06°	4.52°
2	85	172	12.13°	2.08°	6.82°	21.49°	0.62°	2.36°	1.36°	3.90°	5.92°	5.75°	13.11°	13.44°
3	79	164	8.04°	5.63°	15.53°	17.60°	10.01°	6.07°	15.16°	19.10°	8.79°	4.82°	15.54°	20.09°
4	70	167	5.68°	4.41°	0.60°	0.84°	8.47°	1.62°	1.12°	8.88°	8.12°	2.48°	2.30°	7.91°
5	68	168	3.41°	2.17°	1.31°	4.24°	4.52°	2.34°	1.83°	7.59°	5.63°	1.09°	2.10°	7.83°
6	67.6	163	6.43°	2.25°	11.39°	19.76°	10.19°	3.74°	9.78°	23.65°	11.69°	4.41°	9.38°	25.64°
7	67.9	168	8.14°	1.73°	3.70°	13.21°	6.37°	0.73°	5.75°	12.97°	12.15°	1.30°	0.68°	13.81°
8	44	152	5.70°	0.82°	3.28°	8.51°	5.67°	1.40°	4.44°	8.87°	6.59°	1.27°	4.27°	9.11°
9	65	158	15.90°	0.73°	6.24°	21.86°	11.00°	1.31°	7.07°	19.02°	5.40°	2.09°	12.27°	20.18°

**Table A2:** MAD of each one of the four differences at different speeds in all subjects.

			4.0 km/h				4.5 km/h				5.0 km/h			
Subject	Weight (kg)	Height (cm)	A_1_	A_2_	A_3_	A_4_	A_1_	A_2_	A_3_	A_4_	A_1_	A_2_	A_3_	A_4_
1	60	159	1.09°	0.55°	0.70°	0.82°	1.11°	0.68°	1.03°	1.18°	1.42°	1.32°	1.36°	1.50°
2	85	172	2.06°	1.25°	1.74°	1.66°	0.59°	0.70°	0.91°	0.87°	1.13°	0.85°	0.99°	1.02°
3	79	164	0.68°	0.59°	0.94°	0.80°	1.60°	1.71°	0.82°	1.49°	1.24°	0.62°	1.03°	0.78°
4	70	167	0.45°	0.62°	0.56°	0.41°	2.15°	1.23°	0.45°	1.47°	1.33°	1.01°	0.97°	1.36°
5	68	168	2.30°	1.53°	1.23°	1.64°	1.39°	1.73°	1.83°	2.16°	1.89°	1.31°	2.14°	1.82°
6	67.6	163	1.20°	1.92°	0.91°	2.23°	0.91°	1.14°	2.21°	1.67°	1.87°	0.70°	1.27°	0.42°
7	67.9	168	1.21°	1.14°	1.27°	1.41°	1.39°	0.54°	1.22°	2.72°	1.01°	0.47°	0.60°	0.83°
8	44	152	0.50°	0.67°	1.06°	1.57°	0.39°	0.72°	0.57°	0.65°	0.63°	0.46°	0.46°	0.83°
9	65	158	1.76°	0.66°	0.45°	1.29°	1.37°	0.81°	1.64°	1.93°	1.01°	0.71°	1.28°	1.75°
